# Theoretical and Practical Considerations for Combating Mental Illness Stigma in Health Care

**DOI:** 10.1007/s10597-015-9910-4

**Published:** 2015-07-15

**Authors:** Thomas Ungar, Stephanie Knaak, Andrew CH Szeto

**Affiliations:** North York General Hospital, Toronto, ON Canada; Faculty of Medicine, University of Toronto, Toronto, ON Canada; Mental Health Commission of Canada, 320, 110 Quarry Park Blvd, Calgary, AB T2C 3G3 Canada; Department of Psychology, University of Calgary, 2500 University Dr. NW, Calgary, AB T2N 1N4 Canada

**Keywords:** Stigma, Mental health and illness, Mental illness stigma, Healthcare providers, Anti-stigma interventions, Anti-stigma model

## Abstract

Reducing the stigma and discrimination associated with mental illness is becoming an increasingly important focus for research, policy, programming and intervention work. While it has been well established that the healthcare system is one of the key environments in which persons with mental illnesses experience stigma and discrimination there is little published literature on how to build and deliver successful anti-stigma programs in healthcare settings, towards healthcare providers in general, or towards specific types of practitioners. Our paper intends to address this gap by providing a set of theoretical considerations for guiding the design and implementation of anti-stigma interventions in healthcare.

## Introduction

Stigma is a major concern for persons living with mental illnesses. Stigma operates at structural (e.g., discriminatory and/or exclusionary policies and laws), interpersonal (e.g., prejudicial attitudes and behaviors that increase social distance and status loss) and intrapersonal levels (e.g., self-stigma and its associated effects), and involves a deep-seated combination of stereotypes, prejudice and discrimination, which result in social exclusion and status loss for people living with a mental illness (Link and Phelan 2001; Thornicroft 2006). It is a primary vehicle for the entrenchment of discriminatory behaviors, practices and structures, and has been identified as a major barrier to timely and accessible care, recovery, and quality of life (Sartorius and Schulz 2005; Stuart et al. 2012). As such, reducing the stigma and discrimination associated with mental illness is becoming an increasingly important focus for research, as well as for policy, programming, and intervention work (Abbey et al. 2011; Stuart et al. 2012).

One particular area of focus is that of the healthcare sector (Arboleda-Flórez and Stuart 2012; Kassam et al. 2012; Pietrus 2013). While it has been well established that the healthcare system is one of the key environments in which persons with mental illnesses experience stigma and discrimination (Horsfall et al. 2010; Lauber et al. 2006; Ross and Goldner 2009; Thornicroft et al. 2010), there is little published literature on how to build and deliver successful anti-stigma programs in healthcare settings, towards healthcare providers in general, or towards specific types of practitioners. Our paper intends to address this gap by providing a set of theoretical considerations for guiding the design and implementation of anti-stigma interventions in healthcare. Our hope is that these considerations provide a beginning platform from which to direct future empirical investigations and further theoretical development, and that they may also ultimately contribute to improved care and health outcomes of persons with mental illness.

## Why Are Healthcare Providers an Important Target for Stigma Reduction?

Healthcare providers are an important target group for anti-stigma interventions. Healthcare providers are generally caring individuals who make it their work to help others yet stigma has been identified as one of the primary barriers to access care and to receiving equitable quality of care (Abbey et al. 2011; Schulze and Angermeyer 2003; Schulze 2007; Stuart et al. 2012). This can contribute to greater internalization of stigmatizing beliefs and self-silence among persons living with mental illness, inadequate access to proper treatment, less treatment compliance, breakdown of the therapeutic relationship, and greater avoidance of healthcare services (Byrne 2000, 2001; Corrigan 2004; Ross and Goldner 2009; Schulze 2007; Schulze and Angermeyer 2003; Thornicroft et al. 2007). Other aspects of stigmatization include discriminatory behaviors and practices, diagnostic overshadowing, fragmentation and marginalization, and less timely and/or less adequate treatment for non-mental health medical concerns (Atzema et al. 2011; Ross and Goldner 2009; Stuart et al. 2012; Thornicroft 2006, 2008; Thornicroft et al. 2010). These kinds of treatment disparities are believed to account for a substantial proportion of the excess mortality of patients with a mental illness (Druss et al. 2001).

**Fig. 1:**
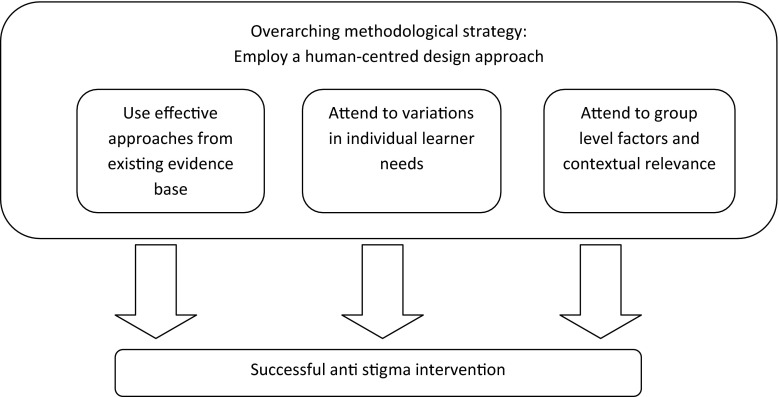
Proposed conceptual model for guiding mental illness anti-stigma programming in health care

In the sections that follow we discuss a number of considerations for the design and delivery of anti-stigma efforts in healthcare. First, we discuss the current evidence and how this applies specifically to healthcare contexts. We then discuss some ways in which attention to both individual and group-level variations in learner needs might be of benefit when designing and delivering anti-stigma interventions. Finally, given the complexity and multi-dimensionality of stigma and its effects, we propose the use of a human centered design approach as providing a promisingly useful overarching methodological strategy for conceiving, designing, delivering, and evaluating anti-stigma programs and efforts, and discuss how such an approach intersects with the Continuous Quality Improvement cycle of ‘Plan-Do-Study-Act’. These considerations may be conceived as fitting together in a general model, such as the one depicted below (Fig. 1).

## Considering the Current Evidence Base

One of the most promising strategies in the evidence-based literature for stigma reduction is that of social contact (Corrigan et al. 2012; Stuart et al. 2012), preferably multiple forms or points of social contact (Knaak et al. 2014), and particularly when Allport’s (1954) four optimal contact conditions (i.e., equal status, cooperation, work towards a common goal, support from authorities) are observed (Couture and Penn 2003; Pettigrew and Tropp 2006, 2008). Contact-based approaches, which involve people with lived experience of mental illness sharing their personal experiences of illness and recovery, can diminish anxiety, heighten empathy, make personal connections, and improve understanding (e.g., Blascovich et al. 2001; Corrigan 2000; Couture and Penn 2003; Pettigrew and Tropp 2008).

There is an interesting quandary in the case of health providers, however, in that they are already in frequent day-to-day contact with persons with mental illnesses. If contact is supposed to be effective at reducing stigma, why do healthcare providers still manifest stigmatizing attitudes and behaviors? One possibility is that most of the contact health professionals have with persons with mental illnesses are at times when patients are at their most unwell. It is believed that this may give many health providers a biased view of mental illness, especially concerning the likelihood of recovery (Hugo 2001; Stuart et al. 2012; Thornicroft 2006). Another possibility is that the nature of the contact is not typically in keeping with Allport’s (1954) four criteria, particularly equal status between parties. To this end, we suggest that contact-based interventions for healthcare providers strongly emphasize and model recovery as a key part of their message, a recommendation supported by strong recently found empirical support in a meta-analysis of key ingredients for anti-stigma programming in healthcare provider populations (Knaak et al. 2014). Contact-based approaches would also benefit from situating persons with lived experience of mental illness vis-à-vis their audience as peers or ‘client educators’ as opposed to patients. Evaluations of anti-stigma programs in Canada using these criteria have been showing promising results in this regard (Pietrus 2013; see also Patten et al. 2012).

There is also emerging evidence that stigmatization among health professionals may, at least in part, be connected to a lack of skills to comfortably assess, communicate with, and treat persons with mental illnesses (MacCarthy et al. 2013; Ross and Goldner 2009). By enhancing communication skills as well as health provider comfort and confidence, skill-based training may improve the quality of interpersonal contact between health providers and patients, leading to more positive attitudes, diminished social and clinical distance, improved client experiences, and better care. To this end, teaching healthcare providers ‘what to do to help’ is also emerging an important ingredient in anti-stigma programming, particularly when used in conjunction with other identified key ingredients (Knaak et al. 2014). It is an approach that has demonstrated success in Canada (e.g., Knaak and Patten 2013, 2014; MacCarthy et al. 2013), although more research is still required.

## Considering Variations in Individual Learner Needs

In developing effective anti-stigma interventions, developers also need to take into consideration other factors, such as individual learner needs, to help enhance the efficacy of their program and approach. A core tenet of education theory is to ‘start with the learner’ (Attard et al. 2010). Understanding where individual healthcare providers ‘are at’ in terms of learning style and program delivery preferences (Kolb 1985; McCombs and Whisler 1997), as well as their existing attitudes and behaviors about mental illness and towards persons with mental illness, is an important consideration for effective anti-stigma programming. Of particular interest is the distinction between perceived and unperceived learning needs (Myers 1999; Ratnapalan and Hilliard 2002) Indeed, an ongoing challenge is that healthcare providers often do not recognize or believe that their behaviors and attitudes towards patients with mental illnesses are stigmatizing and/or discriminatory (Arboleda-Flórez and Stuart 2012). As such, we propose that stages of change theory (see Norcross et al. 2011; Prochaska et al. 1992) may offer a useful conceptual tool in this regard. Although this model is not without criticism in the context of clinical care (e.g., West 2005; DiClemente 2005), it remains a practically useful and widely favored approach for understanding the continuum of learning and behavior change (Noar et al. 2007; Sullivan 2011), and has been employed with some success in other areas of medical education (Shirazi et al. 2009; see also Buckley et al. 2003; Gask 2013).

Shirazi et al. (2009), for example, successfully demonstrated the use of a stages of change model to promote physical activity and improve adherence with strength and balance training recommendations at levels sufficient to prevent osteoporosis in Iranian women aged 40–65 years. In this study, participants received tailored programming based on how they scored on a ‘stages of exercise change’ questionnaire. Results showed that individuals in the training group had a positive, significant progression along the stages of change continuum whereas no progression in stages occurred in the control group. Similarly, Buckley et al. (2003) used a stages of change approach as an evaluative tool to assess the impact of a research transfer training course for scientists. They argued that a stages of change model of evaluation measuring changes in participants’ attitudes, intentions and actions provided an enhanced understanding of the program’s impacts by showing changes that would otherwise have been overlooked. They also noted that such an evaluative instrument could also be used as a pre-course needs assessment to tailor a course to the specific change needs of participants.

Using this type of approach health professionals who do not recognize or believe their behaviors and attitudes towards patients with mental illnesses are stigmatizing and/or discriminatory would be considered to have ‘unperceived’ learning needs, and the assumption could be made that they are likely at the pre-contemplation or contemplation stages of change. The main educational goal is primarily one of realization or ‘transformative learning’ (i.e., the expansion of consciousness through the shifting of one’s perspective or worldview) (Clark 1993; Mezirow 1991; Ungar 2012). To this end, the use of tools like implicit attitude measures (e.g., Norman et al. 2010; Rüsch et al. 2010; see also Project Implicit Mental Health 2014) could prove useful, not only to better identify early stage learners, but also for helping early stage learners to recognize and become more aware of their own prejudices and negative behaviors. Gawronski et al. (2007) have suggested that implicit attitude measures can be important as they have been demonstrated to predict more spontaneous and less controllable behaviors not generally predicted by “traditional” explicit attitudinal measures (e.g., Asendorpf et al. 2002; Dovidio et al. 1997). A possible implication is that by identifying negative implicit biases in early stage learners, they can recognize that despite having control of some behaviors, they may still be susceptible to behaving in a negative manner towards those with mental illnesses (e.g., gaze aversion).

Strategic program marketing may also be a useful strategy for attracting early stage learners. Indeed, achieving desired levels of program attendance remains an ongoing challenge for many anti-stigma programs directed at healthcare providers (Pietrus 2013; Weinerman 2012). The second author, in personal discussions with anti-stigma programs in Canada, learned of an example where a program was marketed two different ways, resulting in two different participation outcomes. For the first offering, the program was promoted as an anti-stigma intervention. Turnout was low. For the second offering, it was renamed and marketed as a program providing education on violence and mental illness. The result was a high level of participation. In as much as strategic marketing may be helpful in maximizing program participation, it would also be important to ascertain whether different ways of program marketing differentially impact outcomes.

There are also many healthcare providers at the other end of the learning spectrum, including many who are already proactive in anti-stigma programming and intervention work (the first author is one such example). While some of these individuals readily welcome interventions designed to combat stigma towards mental illness (Roberts and Bandstra 2012), for others there may be a sense of ‘talking to the converted.’ Qualitative feedback from program evaluations in Canada show that some participants feel their participation in anti-stigma training accomplishes little to change their attitudes or behaviors, as they say they are already well attuned of the problem of stigma and already treat all persons with mental illness with great compassion and care (e.g., Knaak and Patten 2013, 2014). In many of these instances, participants say the program provided a refresher or reminder of something they already knew as opposed to any real shift in perceptions or behavioral intentions, suggesting these learners may be closer to the maintenance phase. For healthcare providers further along the continuum of change, they would likely benefit more from a heavier focus on actionable strategies, as well as opportunities for advocacy, leadership and/or championing of ongoing anti-stigma efforts, including quality improvement and policy change work (Arboleda-Flórez and Stuart 2012).

In order to better develop and understand health provider variations in the context of a continuum of change model, a particularly fruitful area for future research is the development of measures that can adequately assess healthcare providers’ perceptions of stigma and healthcare behaviors, allowing different kinds of interventions to be strategically applied to the most suitable learners. As far as we are aware, such measures do not yet exist. Effective programming begins by gauging an individual’s current behavioral ability, followed by sorting those individuals into relevant learner groups and offering a combination of approaches and marketing techniques to target differing levels of awareness, knowledge, and readiness to change (Gask 2013).

## Considering Group Level Factors

Variations also exist at the group level—homogeneity across occupations, departments or disciplines cannot be assumed. A recent survey of healthcare providers in Canada, for example, found differing levels of stigma across six major physician groups, including psychiatrists, family physicians, surgeons, anesthetists and others (Bird 2012). Using a validated measure, the Opening Minds Stigma Scale for Healthcare Providers (OMS-HC) (Kassam et al. 2012; Modgill et al. 2014), this research found the highest stigma ratings among surgeons, followed closely by anesthetists and emergency rural physicians. This was followed next by family physicians. Psychiatrists had the lowest stigma ratings. Other research comparing attitudes across various health professions, as well as between practicing providers and students, has also found considerable variation (Lauber et al. 2004; Magliano et al. 2004).

It is well argued that knowledge or information must be embedded in its own context to have meaning for learners (Brown et al. 1989; Graham et al. 2006; Jacobson et al. 2003; Lave and Wenger 1991). As such, anti-stigma interventions should be designed, delivered, and incented so as to address the specific interests, learning needs and characteristics of the specific participant group, including considerations of local organizational culture and context and the workings of the ‘hidden curriculum’ (Marsh and Willis 2007). This suggests there may be considerable value in taking a more ‘emic’ approach to thinking about anti-stigma programming, as it this could allow programs to leverage knowledge about the local organizational culture and the real world of practice of the particular group, organization, or department being targeted (Arboleda-Flórez and Stuart 2012; Marsh and Willis 2007; Ungar and Knaak 2013a). Taking a more emic approach—by identifying/training key opinion leaders and trainers from within one’s organizational group or department, for example—may also enhance the likelihood that the program will be valued and trusted by the learner group, and its key messaging/learnings more eagerly adopted (Davis 1998).

Additionally, the same tool, model, wording or approach may carry different meanings in different contexts, settings and/or among different target groups (Brown et al. 1989; Lave and Wenger 1991). Hospital emergency departments and community primary care practices, for example, tend to see clients at different stages of illness acuity and severity, are likely to have different levels of client contact and treatment objectives, and will have different types of work processes and flows as well as remuneration incentives. They may also have different explanatory schemas or beliefs about how mental illness can and should be treated in these environments (Gask 2013).

The question of healthcare providers’ differing understandings of mental illness is an interesting consideration for understanding learner group needs. In as much as we know that different cultural groups have different ways of explaining and making sense of mental illness (Haslam et al. 2007; Kleinman 1980), anti-stigma efforts are likely to be more successful if they acknowledge, understand, and seek change from within the target group’s existing schema (Gask 2013; Ungar and Knaak 2013b). While existing research suggests that emphasizing biological aspects of mental illness does not reduce stigma and discrimination among the general public (e.g., Pescosolido et al. 2010; Schomerus et al. 2012), the same cannot be assumed for health professionals. Medical practitioners are in the specific business of discovering, fixing, treating, and controlling biologic disorder; they are healers of the somatic. As such, they apply a different set of cognitive interpretations/judgments (i.e., less essentialist, greater capacity for recovery) to a biomedical view of mental illness than does the general public. Using biological information to emphasize the “bio” components of biopsychosocial mental illnesses may help to shift the conception of those illnesses from something functional (i.e., not real) to something organic (i.e., real) (Ungar and Knaak 2013b). Although such an approach may not break down the dualistic mind–body mindset (Thomas 2013), leveraging health providers’ existing paradigm may nevertheless be productive as an initial intervention. This kind of strategy would also be consistent with research showing that persons with lived experience of mental illness tend to favor a biomedical interpretation of their illness as a stigma management strategy (Schreiber and Hartrick 2002).

Additionally, although anti-stigma interventions often favor a more generalist approach—by targeting mental illness as a whole as opposed to specific disorders—it is important to note that different forms of mental illness also invoke different explanatory schemas (Haslam and Giosan 2002; Szeto et al. 2013). There is also emerging evidence to suggest that healthcare providers hold different explanatory schemas for different forms of mental illness (Gask 2013). This thus presents another important consideration for tackling stigma among healthcare providers, especially if the interest is to target stigma against a particular form of mental illness.

## Methodological Strategy

In as much as the considerations described above are believed to be important for stigma reduction within healthcare contexts, an overarching methodological strategy to help guide and inform the design process is still needed. Healthcare traditionally uses technical-scientific, reductionist methods to understand and address problems—isolating things to their smallest part and studying them in relative isolation. But these familiar ways may be limited in their ability to fully allow us to understand and address the complex social phenomenon of stigma. We believe that humanistic methods of inquiry, which focus heavily on such ethnographic elements as attitudes, behaviors and culture, to be particularly crucial.

To this end, we propose the use of a human centered design approach as a potentially useful strategy, allowing also for a productive balance of both technical-scientific and humanistic inquiry. Human centered design is not a new approach per se, but rather a general methodological orientation encompassing several methods increasingly used within and outside healthcare. These include Human Factors (Vicente 2006; Sawyer 1996), Empathic Design (Leonard and Rayport 1997) and Design Thinking (Brown and Kātz 2008; Martin 2009). Human Factors was initially described with a focus on the design of medical devices and ergonomics (Sawyer 1996). Human Factors is also used when looking at medical errors for quality improvement. Empathic Design is a set of techniques from the field of business used to aid in understanding customer and user needs. It emphasizes observation of behaviors in the user’s own context and environments. It also emphasizes the discovery and consideration of unarticulated and unperceived customer/user needs, motivations and behaviors (Myers 1999; Ratnapalan and Hilliard 2002). Design Thinking promotes the idea that ‘thinking like a designer’ can transform the way we develop strategies, products, or services. It conceives of design not as a late stage decorative or aesthetic activity, but as using innovative and creative thinking from the outset, at the strategic level.

Human centered design methods have a number of features and capacities in common, which we believe make them well suited to the challenges and complexities of designing and delivering successful and effective anti-stigma interventions in healthcare. They include: ecological validity; human centeredness and empathy; curiosity, optimism and experimentation; and collaboration.

Ecological validity refers to the fact that human action is situated and is contingent on contextual factors/variables. In this view, “to obtain ‘valid’ results, humans should be studied in the richness of their natural environment” (Soegaard 2006: 1). When planning anti-stigma interventions, an emphasis on ecological validity may mean including members of the target audience from the outset, in core strategic roles as opposed to tactical or token late stage approval/sign off roles. It also means including persons with lived experience of a mental illness in all phases of the process, as the end goal is always directed towards improvements in their experiences, healthcare interactions, and quality of care. Ecological validity emphasizes the importance of intervention prototype refinement in the ‘real world’ of the end user (see also Sartorius and Schulz 2005; Stuart et al. 2012) allowing program planners to discover lessons learned and to self-correct quickly, limiting potentially costly errors or omissions that may be crucial to participation or program efficacy, before wider implementation and full cost.

### Human Centeredness and Empathy

Approaching stigma as a complex social process requires an approach that emphasizes understanding why an individual, group or culture may manifest stigma. This is different than an approach which sees the individual or group as non-compliant, wrong, or somehow not behaving as they ‘should’. Instead of ignoring or fighting against ingrained beliefs and cultural norms, a human centered approach allows anti-stigma intervention planners to accommodate, strategically collude and otherwise leverage (Ungar and Knaak 2013a) these in the design process, to achieve desired outcomes. For example, it may be useful for understanding the stigmatizing behavior of avoidance/social distance—and therefore how to effectively combat it—to learn if a person is fearful of violence, to learn if they simply doesn’t know ‘what to say’ or ‘what to do’, or to learn if it another combination of feelings and beliefs driving the behavior. Empathy and human centeredness is also crucial to understanding the experiences of patients and their families as they relate to stigma, as these represent the core start and end points of any anti-stigma effort or program.

### Curiosity, Optimism and Experimentation

Human centered design approaches share a belief that there is always a solution, or at least an improvement, from the current state. There is a tolerance for experimentation, rapid prototyping, playfulness, wild ideas, brainstorming, and changing direction. Using a human centered design approach, an anti-stigma intervention would first be prototyped, then redesigned based on observation and user feedback and evaluation. This pre-implementation prototyping phase allows for early rapid changes, as well as customization and openness to the unpredictable—before investment of the resources of full implementation. This flexibility is a contrast to much of traditional project management, which tends to follow a more fixed or phase-by-phase process whereby projects are designed and implemented over a predetermined period of time followed by an evaluation phase, and where only at completion is there a full consideration of what was worked and what did not.

### Collaboration

Human centered design approaches all emphasize collaboration as key to successful design. This includes using interdisciplinary, cross-disciplinary or trans-disciplinary teams, and including the voices and perspectives of persons with lived experience of a mental illness at all stages of the process. For the design of anti-stigma programs and interventions, it also means giving thought to using broad planning and implementation teams which may also include such forms of expertise as professional marketers, cultural anthropologists, arts performers, etc.

As well, an essential component of any improvement agenda is determining whether the intervention leads to the desired change(s) and if such changes result in better outcomes. In this vein, the Continuous Quality Improvement (CQI) method of ‘Plan-Do-Study-Act’ (PDSA) offers a useful base from which to work. The PDSA cycle in particular provides a guide for testing a change in the real work setting—by planning it, trying it, observing the results, and acting on what is learned (Langley et al. 2009; Taylor et al. 2013).

In many respects, a human centered design approach adopts a similar lens to that of CQI, which also capitalizes on the collective expertise of all stakeholders to figure out ways to solve problems together and which targets the processes involved in day-to-day operations at both administrative and service delivery levels, all with the primary end goal of improved quality of care (Vicente 2006). Both human centered design and PDSA allow for iterative real world local context testing and openness to both quantitative and qualitative evaluation for learning in complex systems such as healthcare. The main difference between the PDSA model and human centered design is our emphasis on the importance of recognizing that that new ideas, processes or strategies do not necessarily follow a linear logic nor emerge from focusing on enhancing an existing model. Human centered design also directs considerable energy and attention to the human elements of a quality improvement, which may include such aspects as attitudes, interests, personal or professional motivations, human error, human limitations, as well as group dynamics and culture.

If one were to apply the PDSA framework to a human centered design approach, for example, an additional planning stage (i.e., ‘Pre-Plan’) would be included in the cycle, whereby broad yet rich ethnographic inquiry allows one to get a fuller and more complete understanding of the problem at hand. As well, in the ‘Plan’ stage, human centered design would encourage the use of intuitive or abductive logic and ‘outside the box’ ideas from as many different and collaborative cross disciplinary viewpoints as possible—in addition to the usual analytic methods for idea generation (Brown and Kātz 2008; Martin 2009).

Human centered design would move to the ‘Do’ and ‘Study’ steps of the PDSA cycle with prototyping and rapid engagement in an iterative series of low cost, low investment in vivo cycles of ‘Do-Study-Replan-Redo-Restudy-Replan,’ repeating as many times as needed until the desired outcome or quality improvement is demonstrated, including business sustainability and viability of the intervention prior to full implementation. Similar to PDSA, the ‘Act’ stage of human centered design would include broader implementation along with greater investment of resources and dissemination of a design for quality improvement.

As implied by both human centered design and the PDSA cycle, evaluating the outcomes of programs and using these outcomes as foundations for a ‘redo’ or program improvement is a part of the necessary implementation process. However, this evaluation component is often neglected or improperly conducted within the context of programs with the goal of stigma reduction (Szeto and Dobson 2010). Although anti-stigma program designers may have good intentions, good intentions may results in little or no stigma reduction [see the meta-analysis by Griffiths et al. (2014)]. At worst, programs could actually increase stigma towards those with mental illnesses. Therefore, it is important to assess stigma outcomes via validated tools (e.g., Kassam et al. 2010; Modgill et al. 2014) to ensure the program does exactly what it was intended to do. Although the evaluation of programs is a vast topic in itself and beyond the current scope, literature does exist to describe the evaluation process for anti-stigma programs (e.g., Szeto and Dobson 2014) using standard evaluation methods, such as the Kirkpatrick model (Kirkpatrick 1994).

## Conclusion

Combating stigma in healthcare is no easy task. The reality of often-already-overscheduled work lives, multiple and competing demands for in-service and training, and resource constraints for program funding and/or to provide for relief staff are a few of the many challenges facing anti-stigma programmers. Additionally, we know that effectively combating stigma ultimately requires multi-dimensional, multi-level approaches that address stigma holistically, from programming to structural change (Link and Phelan 2001; Roberts and Bandstra 2012; Stuart et al. 2012). As we have discussed, taking a targeted and multi-level approach to stigma reduction is much preferred over a ‘one size fits all’ generalist model, as explanatory schemas, group needs, contextual realities, and individual educational and skill needs vary.

We have attempted to highlight that what drives stigma may be different for different groups and different individuals. Our specific considerations, along with the suggestion of adopting a guiding strategy informed by principles of humanistic design methods, are meant to provide the beginnings of a conceptual model to help structure further thinking and research questions about reducing stigma in healthcare environments, and improving the quality of care and therapeutic experiences of persons with lived experience of mental illness. While our primary interest here has been considerations for stigma reduction specifically in the context of healthcare, many of the concepts may well be applicable for other target groups. Ultimately, it is our hope that the ideas discussed here provide a launching pad for further investigation and development, with the ultimate goal of improved interactions and quality of care for persons with living with mental illnesses.
